# Predictors of Early Cardiac Implantable Electronic Device Lead Dislodgement in the Elderly

**DOI:** 10.3390/ijerph192214766

**Published:** 2022-11-10

**Authors:** Rafal Mlynarski, Agnieszka Mlynarska, Michal Joniec, Sylwia Gladysz-Wanha, Maciej Honkowicz, Joanna Stachanczyk, Krzysztof S. Golba

**Affiliations:** 1 Department of Electroradiology, School of Health Sciences, Medical University of Silesia, 40-635 Katowice, Poland; 2 Department of Electrocardiology, Upper Silesian Heart Centre, 40-635 Katowice, Poland; 3 Department of Gerontology and Geriatric Nursing, School of Health Sciences, Medical University of Silesia, 40-635 Katowice, Poland; 4 Department of Electrocardiology and Heart Failure, School of Health Sciences, Medical University of Silesia, 40-635 Katowice, Poland; 5 Department of Cardiac Rehabilitation, Murcki Hospital, 40-749 Katowice, Poland

**Keywords:** CIED implantation complications, lead dislodgement, elderly, frailty

## Abstract

Introduction: One of the most frequent cardiac implantable electronic device (CIED) implantation complications is lead dislodgement, especially in the older adult population. Little evidence is available about the influence of frailty on the risk of lead dislodgment after CIED implantation procedures; thus, the evaluation of frailty could be relevant for the course and safety of the implantation procedure, especially among the elderly with cardiovascular diseases. This study aimed to assess the risks and predictors of early lead dislodgement in the elderly population. Methods: Between 2008 and 2021, 14,293 patients underwent implantations. In 400 elderly patients, lead dislodgement was confirmed, and frailty was retrospectively calculated. Results: The most frequent dislodgement according to the lead position was that of the atrial lead (133; 33.3%). In the logistic regression, frailty (OR: 1.8196, 95% CI:1.4991–2.2086; *p* < 0.0001) and age (OR: 1.0315, 95% CI:1.0005–1.0634; *p* < 0.0461) were independent predictors of early dislodgement. In the female group, frailty (OR: 2.1185, 95% CI: 1.5530–2.8899; *p* < 0.0001) was an independent predictor of early dislodgement. Similarly, in the male group, frailty (OR: 1.6321, 95% CI:1.2725–2.0934; *p* < 0.0001) was an independent predictor of early dislodgement. Conclusion: Lead dislodgement often occurs in the elderly. Frailty in both men and women is a predictive factor of early lead dislodgment. Evaluating frailty may be an essential element of proper selection, especially in the elderly undergoing CIED procedures, and, consequently, it could help prevent further complications.

## 1. Introduction

The constantly growing number of cardiac implantable electronic device (CIED) procedures, including the implantation of cardiac pacemakers (PM), implantable cardioverter-defibrillators (ICD), and resynchronization systems (CRT), is related to the aging of the population and the improvement of medical care. Lead technology has recently shown substantial progress, with a reduction in the number of implanted passive leads in favor of leads with active fixation [1,2,3]. Despite the benefits of lead technology, the number of CIED complications is in line with the number of implantation devices [4,5]. One of the most frequent CIED complications is lead dislodgement, which occurs in 2% [6] of cases and is associated with re-operation and following lead fixation [7]. Furthermore, Prutkin et al. [8] showed that lead dislodgement was one of the most substantial risk factors for CIED infection. Early lead dislodgement prolongs hospitalization time and increases patient discomfort [9]. The reasons for lead dislodgement can be spontaneous or related to additional procedures. The data from REPLACE Registry showed that 7.9% of lead dislodgements in patients were in those undergoing device upgrades or pocket revision [10,11]. Another type of lead dislodgement is atrial or ventricular perforation caused by CIED leads. This is rare but potentially life-threatening [2,6,12]. Lead dislodgements can also result in complications, including cardiac tamponade, cardiac arrest, pneumothorax, and pacing failure, leading to hemodynamic instability and sudden death [11,13]. Moreover, there is no clear evidence of how age influences the risk of lead dislodgement in older patients. An essential part of biological age is frailty, which is a predictive factor often used in gerontology and cardiology and which may play a key role in electrotherapy [14]. Little evidence is available about the influence of frailty on the risk of dislocation after CIED implantation procedures, thus the evaluation of frailty could be relevant for the course and safety of the implantation procedure, especially among the elderly with cardiovascular diseases (CVDs) [15]. Therefore, this study aimed to assess the number of lead dislodgements in the elderly population and to analyze the risk factors that might affect early dislodgment occurrence.

## 2. Materials and Methods

**Figure 1:**
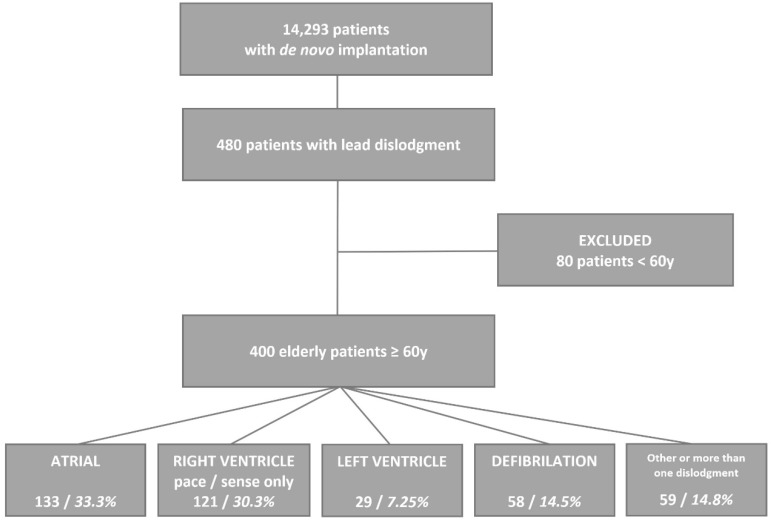
Study design and patient selection.

A total of 14,293 patients were hospitalized between 2008 and 2021 in the Electrocardiology Department for de novo CIED implantation, including PM, ICD, and CRT. The data of 480 patients with lead dislodgement were entered into the retrospective registry, and a group of elderly patients aged 60 or older was selected. The study design and patient selection are summarized in a flowchart in Figure 1.

Arterial hypertension was the most common comorbid disease (84.1%) in the enrolled patients. The next most common comorbidities were ischemic heart disease (56.9%), atrial fibrillation (41.6%), heart failure (36.6%) and diabetes (30.4%). Most patients received ACE blockers (83.4%), B-blockers (74.8%), calcium blockers (70.3%) and statins (67.1%).

Episodes of lead dislodgement data were divided into two categories: early (up to 30 days from implantation) and late (more than 30 days from implantation). The existence and intensity of frailty in the analyzed patients were calculated retrospectively.

### 2.1. Frailty Evaluation

Frailty was retrospectively based on data before CIED implantation evaluated using the 7-point Canadian Study of Health and Aging Clinical Frailty Scale (CSHA-CFS). This scale has good predictive validity (regarding death and the need for institutional care), and its predictive power relies on clinical judgment. Values = 4 described patients who might be in danger of developing frailty. Patients with values ≥ 5 were recognized with as having frailty. The detailed description of the individual points is follows, according to The Canadian Study of Health and Aging Clinical Frailty Scale [16]:

1. Very fit—Robust active, energetic, well-motivated, and fit; these people commonly exercise regularly and are in the fittest group for their age.
2. Well—Without any active disease, but less fit than people in category 1.
3. Well, with treated comorbid disease—Disease symptoms are well controlled compared to those in category 4.
4. Apparently vulnerable—Although not clinically dependent, these people commonly complain of being “slowed down” or having disease symptoms.
5. Mildly frail—With limited dependence on others for the instrumental activities of daily living.
6. Moderately frail—Help is needed with both the instrumental and non-instrumental activities of daily living.
7. Severely frail—Completely dependent on others for the activities of daily living or terminally ill.

### 2.2. Statistical Analysis

In order to check the normality of the data distribution, the Shapiro–Wilk test was used. Logistic regression analysis was used to predict factors responsible for lead dislodgement. The ROC curve analysis was performed to assess the value of frailty to predict a higher risk of dislodgement.

The results were considered significant at *p*-values < 0.05. All the presented analyses were performed using MedCalc^®^ Statistical Software version 20.114 (MedCalc Software Ltd., Ostend, Belgium).

### 2.3. Bioethical Consideration

Participation in this study was anonymous and voluntary. The Local Bioethics Committee of the Medical University of Silesia approved the study protocol (PCN/CBN/0022/KB/8/22). When the study was designed, the protocol complied with the current version of the Helsinki Convention.

## 3. Results

**Figure 2:**
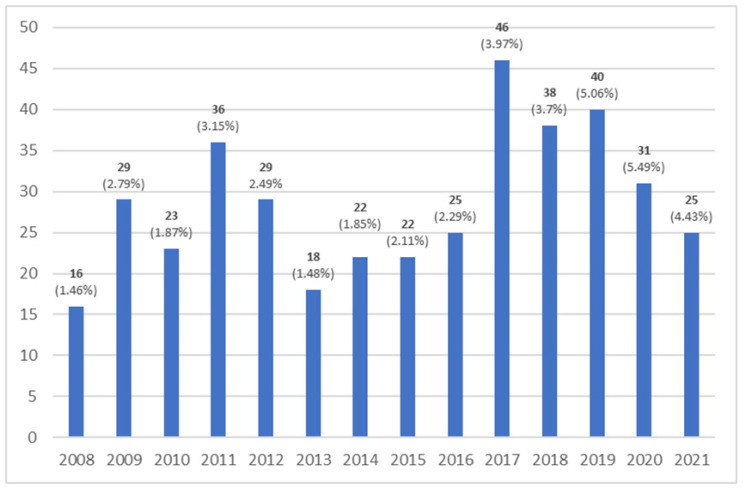
Annual number of dislodgements.

In 400 elderly patients (187 women; aged 74. 0 ± 7.9 for a fourteen-year follow-up), one or more lead dislodgements were noted, and a lead correction procedure was also performed. The exact annual number of dislodgements is presented in Figure 2. In 361 patients, one lead dislodgement was found (90.3%); in 37 patients, two leads were dislodged (9.3%), and three leads were dislodged in two patients (0.5%). A total of 67.5% of devices with dislodgment of the lead(s) were cardiac pacemakers, 12% were implanted cardioverter defibrillators (ICD), 18% were cardiac resynchronization with implanted cardioverter defibrillators (CRT-D) and 2.5% were cardiac resynchronization devices (CRT-P).

The frequency of dislodgement according to the lead position was as follows: atrial lead (133; 33.3%), right ventricle pace/sense only (121; 30.3%), and left ventricle (29; 7.25%). Defibrillation lead dislodgement was found in 58 (14.5%) cases. Additionally, the His pacing lead was dislocated in 13 patients (3.3%). In five patients (1.3%), the temporary pacing lead was dislodged, and in one case (0.3%), the left bundle branch pacing lead was dislodged. In all other cases, two or more leads were dislodged.

In the logistic regression, frailty (OR: 1.8196, 95% CI:1.4991–2.2086; *p* < 0.0001; Nagelkerke R2 = 0.1635) and age (OR: 1.0315, 95% CI:1.0005–1.0634; *p* < 0.0461; Nagelkerke R2 = 0.1635) were independent predictors of early dislodgement. Detailed regression results are presented in Table 1.

**Figure 3:**
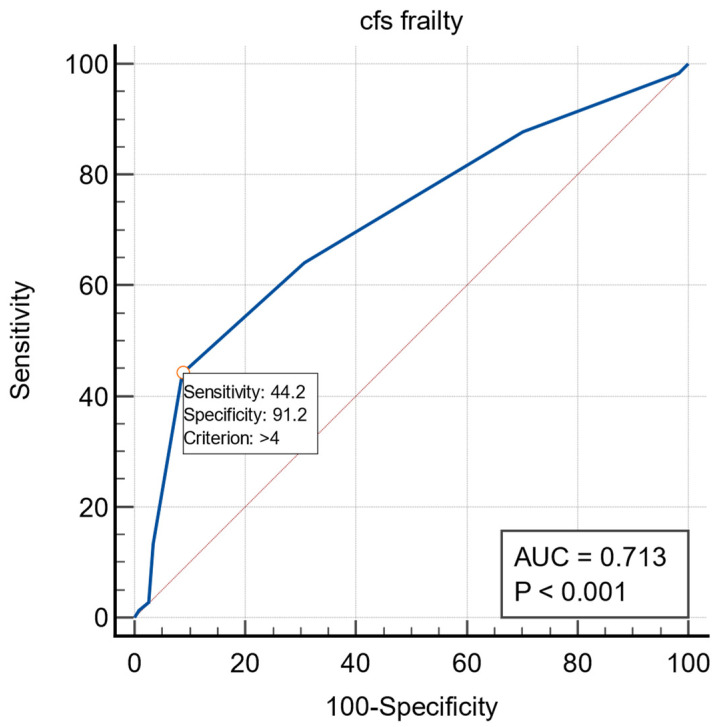
The ROC curves for frailty and dislodgement.

The ROC curves for frailty and early dislodgement are presented in Figure 3. The area under the curve was 0.714 (95% CI:0.667–0.758). The cutoff value for recognizing frailty was > 4 (*p* < 0.0001).

In the logistic regression in the female group, frailty (OR: 2.1185, 95% CI: 1.5530–2.8899; *p* < 0.0001; Nagelkerke R2 = 0.2214) was an independent predictor of early dislodgement. Detailed regression results are presented in Table 2.

**Figure 4:**
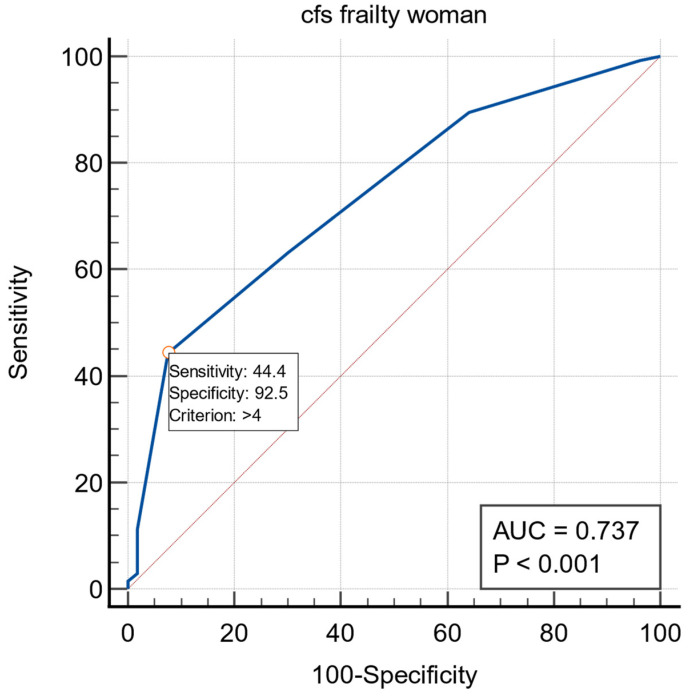
The ROC curves for frailty in the dislodgement in female subgroup.

The ROC curves for frailty and early dislodgement are presented in Figure 4. The area under the curve was 0.737 (95% CI: 0.668–0.799). The cutoff value for recognizing frailty was >4 (*p* < 0.0001).

The same observation in the male group was performed. In the logistic regression, frailty (OR: 1.6321, 95% CI: 1.2725–2.0934; *p* < 0.0001; Nagelkerke R2 = 0.1218) was an independent predictor of early dislodgement. Detailed regression results are presented in Table 3.

**Figure 5:**
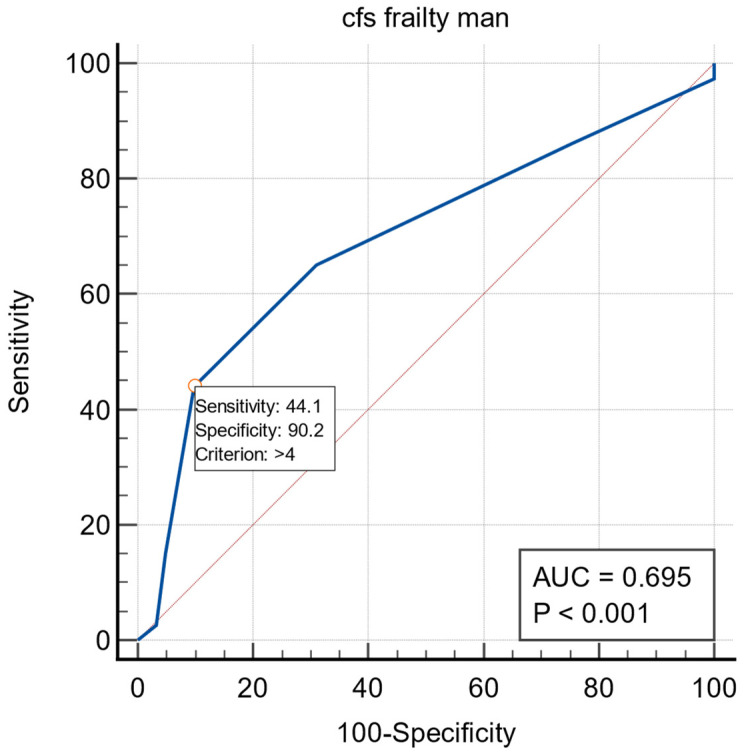
The ROC curves for frailty in the dislodgement in male subgroup.

The ROC curves for frailty and early dislodgement are presented in Figure 5. The area under the curve was 0.695 (95% CI: 0.629–0.756). The cutoff value for recognizing frailty was 4 (*p* < 0.0001).

## 4. Discussion

Increasing use of CIEDs is associated with increased complications, including lead dislodgements. Recently, numerous studies have evaluated the risk factors of lead dislodgements [6,13,17,18]. For instance, Dębski et al. [17] showed that atrial lead position is an independent risk factor of lead dislodgement. Moreover, the authors indicate that the younger the age at implantation, the higher the probability of lead failure (odds ratio: 0.985; *p* < 0.001) [17]. Furthermore, Ghani et al. [6] presented an analysis of 3909 implantation leads and showed that right atrial and ICD lead dislodgements are more frequent than right ventricular lead dislodgements. Moreover, they report that lead dislodgements were more often observed in resynchronization systems with ICD and dual-chamber cardioverter-defibrillators than single-chamber PMs. The MOST trial [18] showed that the most common complication is dislodgement of the right atrial lead (1.7%) in the first 30 days after implantation.

In a subgroup analysis of the MIRACLE ICD study, Cheng et al. reported that older age, female sex, atrial fibrillation, and left ventricular leads are linked to a higher rate of acute lead dislodgement [13]. Furthermore, the authors showed that females had higher complication rates (95% CI: 0.98 to 1.99, *p* = 0.06) but without specifying dislodgements. It is known from the literature that the implantation of CIEDs in advanced age is an independent risk factor for in-hospital mortality [19,20]. Thus, the indication and need for intervention should be determined carefully, particularly in elderly patients. In this report, sex, age, and frailty were independent predictors of early lead dislodgement. However, data on the effect of the frailty syndrome on the occurrence of lead dislodgement are scarce. Frailty is a health condition associated with the aging of the population [21].

Moreover, frailty is frequently overlooked and identified with comorbidities. It is a state of limited capacity reserve of essential systems and their homeostasis. Frailty is caused when chronic inflammation leads to kinesiophobia and further progression of cardiovascular, immune, musculoskeletal, and hematopoietic failure; it results in increased morbidity and mortality [22]. A study by Mlynarska et al. demonstrated that frailty syndrome has a negative impact on the quality of life of patients who qualify for the implantation of CIEDs [23]. Frailty has also been identified as a predictor of poor CRT therapy outcomes [24] and predicts the maintenance of sinus rhythm in the elderly after cardioversion [25].

Furthermore, it has recently been shown that a higher frailty syndrome score is observed in the elderly with accompanying cardiac arrhythmias [26]. Therefore, our results showed the risk of early lead dislodgement by calculating the severity of frailty. Evaluating the occurrence of frailty may be an essential element of the proper selection of elderly patients to undergo CIED procedures. This could help prevent further complications and improve clinical outcomes. In addition, results noted in our study provide insight into carrying out better and faster follow-ups after CIED implantation, especially among patients with high frailty rates. Moreover, studies show that it is necessary to avoid the inadequate lead position during CIED implantation due to the high risk of perforation, especially in the right ventricle’s apex and the right atrium’s lateral wall [6]. In earlier studies, attempts have been made to establish [27] the benefits of implanting single-lead atrioventricular pacemakers performed without fluoroscopy. This report showed that using single-lead VDD pacemakers with electroanatomic navigation systems (The NavX system) would allow dependable visualization of the implantation site. Thus, our study provides the framework for future studies to assess another implantation techniques and choose the most satisfactory option for the elderly. Recently, Pagan et al. [28] presented evidence suggesting that leadless pacemakers are safe alternatives with low rates of procedure-related complications for old, frail patients. In this context, further investigations of appropriate implantation techniques, lead construction technology, and diagnosis of frailty may ensure a high success rate in all patients, especially among the elderly.

Important problems which should be answered is to distinguish between micro and macro dislodgments. Chest radiography allows us to find true, massive dislocations (visible in the radiological assessment), but there is no definition of micro-dislocation, which is often difficult to find. This lack of explicit criteria resulted in the lack of such analyses in the presented paper.

Significant problems related to the implanted cardiological leads are, e.g., too early patient agitation after implantation with psychomotor agitation and inappropriate movements of the limbs and chest, traumatic events, and manipulations in the area of the CIED which can cause possible Twiddler’s syndrome. All of these can potentially be responsible for lead(s) dislodgment. In older adults with frailty syndrome, early autonomous mobilization by the patient or alterations of CIED due to weight loss should also be analyzed. This is especially important in the case of patients at risk of lead dislodgment. Based on the presented analysis, it seems that older adults qualifying for CIED implantation should be especially cared for to prevent lead dislocation.

## 5. Conclusions

In just over 14,000 CIED procedures performed in the past 14 years, the most frequent dislodgement observed was atrial lead dislodgement. Additionally, we report that the incidence of lead dislodgement is high in the elderly. Frailty in both men and women is predictive of early lead dislodgment. Thus, our findings show that the estimation of the prevalence of frailty could be included in routine CIED management to enable high clinical and procedural success.

## 6. Limitations

The main limitations of the study were the single center and the study’s observational nature. No data were collected on implantation techniques leading to lead dislodgements. In addition, the regression models do not include adjustments for other factors beyond sex and age.

## Figures and Tables

**Table 1:** Detailed results of logistic regression.

Variable	B	SE	Wald	95% CI	*p*
Frailty	0.5986	0.09885	36.6727	1.4991–2.2086	<0.0001
Age	0.0310	0.01554	3.9775	1.0005–1.0634	0.0461
Constant	−3.5227	1.20654	8.5248		

B—regression equation coefficient, SE—standard error.

**Table 2:** Detailed results of logistic regression—female subgroup.

Variable	B	SE	Wald	95% CI	*p*
Frailty	0.75069	0.15842	22.4536	1.5530–2.8899	<0.0001
Age	0.040446	0.022820	3.1414	0.9957–1.0889	0.0763
Constant	−4.73957	1.83476	6.6730		

B—regression equation coefficient, SE—standard error.

**Table 3:** Detailed results of logistic regression—male subgroup.

Variable	B	SE	Wald	95% CI	*p*
Frailty	0.48990	0.12698	14.8846	1.2725–2.0934	0.0001
Age	0.02426	0.021824	1.2357	1.0005–1.0634	0.2663
Constant	−2.64838	1.64288	2.5987		

B—regression equation coefficient, SE—standard error.
